# Fundus Autofluorescence as a Sensitive Biomarker of Disease Progression in Bietti Crystalline Dystrophy

**DOI:** 10.1016/j.xops.2026.101166

**Published:** 2026-03-19

**Authors:** Huanyu Zhao, Masatoshi Fukushima, Takahiro Hisai, Yan Tao, Sakurako Shimokawa, Kaho Yamamoto, Yoshito Koyanagi, Koh-Hei Sonoda, Takaaki Hayashi, Shinji Ueno, Manabu Miyata, Kaoru Fujinami, Kazushige Tsunoda, Akio Oishi, Yusuke Murakami

**Affiliations:** 1 Department of Ophthalmology, Graduate School of Medical Sciences, Kyushu University, Fukuoka, Japan; 2 Department of Ophthalmology, The Jikei University School of Medicine, Tokyo, Japan; 3 Department of Ophthalmology, Graduate School of Medicine, Hirosaki University, Hirosaki, Japan; 4 Department of Ophthalmology and Visual Sciences, Kyoto University Graduate School of Medicine, Kyoto, Japan; 5 Division of Vision Research, National Institute of Sensory Organs, NHO Tokyo Medical Center, Tokyo, Japan; 6 Department of Medical Genetics, NHO Tokyo Medical Center, Tokyo, Japan; 7 Department of Ophthalmology and Visual Sciences, Graduate School of Biomedical Sciences, Nagasaki University, Nagasaki, Japan

**Keywords:** Bietti crystalline dystrophy (BCD), Fundus autofluorescence (FAF), Hypo autofluorescent area, Biomarker, Disease progression

## Abstract

**Purpose:**

To characterize the natural history of Bietti crystalline dystrophy (BCD) and to identify reliable outcome measures for detecting disease progression.

**Design:**

A multicenter, prospective observational study.

**Participants:**

Patients with genetically confirmed BCD carrying biallelic pathogenic *CYP4V2* variants, recruited from 6 academic centers in Japan, with a best-corrected visual acuity (BCVA) of 1.0 logarithm of the minimum angle of resolution (logMAR) or better in ≥1 eye at baseline.

**Methods:**

Multimodal imaging and functional assessments included BCVA, static perimetry (Humphrey 10-2), fundus autofluorescence, and OCT.

**Main Outcome Measures:**

Longitudinal changes in BCVA; hypo-autofluorescent (hypo-AF) area; and static perimetry parameters, such as mean deviation (MD), absolute scotoma points (ASPs), transitional zone retinal sensitivity (TZRS), and central subfield thickness (CST).

**Results:**

Thirteen patients with genetically confirmed BCD were followed prospectively over a median of 2.5 years (range: 2.5–3 years). Longitudinal analysis showed progression in hypo-AF area (12.0 mm^2^/year, 95% confidence interval [CI]: 7.1 to 16.9, *P* = 0.003), CST (–4.3 μm/year, 95% CI: –6.6 to –2.1, *P* < 0.001), MD (–1.0 dB/year, 95% CI: –1.7 to –0.3, *P* = 0.02), ASP (2.4 points/year, 95% CI: 1.8–3.0, *P* < 0.001), TZRS (–2.1 dB/year, 95% CI: –2.8 to –1.4, *P* < 0.001), and BCVA (0.04 logMAR/year, 95% CI: 0.01–0.07, *P* = 0.01). The hypo-AF area showed the biggest annual change rate (21.6%) and highest signal-to-noise ratio (1.10) among all parameters. Linear mixed-effects models revealed that hypo-AF area progression was significantly associated with faster loss of MD (*P* = 0.01) and ASP (*P* = 0.004), but not with BCVA, TZRS, and CST changes.

**Conclusions:**

This prospective natural history study suggests that hypo-AF area may serve as a useful structural measure for detecting disease progression in BCD clinical trials, warranting further validation in larger prospective studies.

**Financial Disclosure(s):**

Proprietary or commercial disclosure may be found in the Footnotes and Disclosures at the end of this article.

Bietti crystalline dystrophy (BCD) is an ultrarare autosomal recessive retinal dystrophy characterized by crystalline deposits in the retina and cornea, progressive degeneration of the retinal pigment epithelium (RPE) and photoreceptors, and corresponding visual decline (Online Mendelian Inheritance in Man 210370).^1^, ^2^, ^3^, ^4^ Patients typically experience progressive visual field constriction and substantial loss of visual acuity, with most individuals reaching legal blindness by their fifth or sixth decade of life. Bietti crystalline dystrophy is caused by biallelic pathogenic variants in the *CYP4V2* gene (Online Mendelian Inheritance in Man 608614), located on chromosome 4q35.1–q35.2, and has a coding sequence of approximately 1.6 kb.^5^ *CYP4V2* encodes a 525-amino acid protein belonging to the cytochrome P450 superfamily. This protein is predominantly expressed in the RPE and plays a key role in fatty acid ω-oxidation.^5^, ^6^, ^7^, ^8^, ^9^ More than 200 pathogenic variants of *CYP4V2* have been identified, among which c.802–8_810del17insGC and p.H331P are the most frequent in East Asian populations, particularly in China, Japan, and Korea.^10^ The global genetic prevalence of BCD is estimated to be approximately 1 in 116 000, corresponding to about 67 000 affected individuals worldwide,^10^ with notably higher prevalence in East Asian populations.^8^^,^^10^, ^11^, ^12^, ^13^

Accurate assessment of BCD progression is crucial for clinical management and clinical trials of potential therapeutic agents. Several retrospective studies have evaluated BCD progression.^14^, ^15^, ^16^ In a cohort of 29 patients followed for a mean of 5.9 years, Han et al reported an annual decline in best-corrected visual acuity (BCVA) of 0.08 logarithm of the minimum angle of resolution (logMAR) and an annual decrease of 1.14 dB in mean deviation (MD) of the visual field.^17^ Zhang et al conducted a meta-analysis of 14 studies including 117 cases and reported an annual decline in BCVA of 0.06 logMAR in patients with BCD.^18^ From a structural perspective, Cheloni et al^19^ studied 28 patients with a median follow-up of 7.7 years and reported that, among OCT parameters, ellipsoid zone measures and choroidal thickness showed the fastest rates of progression, at 2.3% to 3.3% and 0.6% to 1.5% per year, respectively.

Several studies have also investigated the relationship between *CYP4V2* variants and clinical phenotypes in BCD. Rossi et al^20^ studied 15 patients and identified 7 novel *CYP4V2* variants, noting marked clinical variability even among those with the same variant. Halford et al analyzed 20 patients and reported that exon 7 deletions were associated with advanced disease stage.^21^ Murakami et al^22^ followed 62 patients for a mean of 7.1 years and found no significant differences in BCVA decline between patients with the homozygous c.802–8_810del17insGC variant (NM_207352.4) and those with other genotypes.

However, all these studies were retrospective in design, and to date, no prospective studies have evaluated disease progression in BCD. Therefore, we here conducted a multicenter, prospective natural history study to assess both structural and functional progression over a 3-year period. In addition, we investigated the relationships between functional and structural parameters to identify reliable outcome measures for monitoring BCD progression.

## Methods

### Study Design and Ethics Statement

This was a multicenter, prospective observational study conducted to investigate the progression of functional and structural markers in patients with BCD. The study was carried out at 6 academic hospitals in Japan: Kyushu University Hospital, Jikei University Hospital, Nagoya University Hospital, Kyoto University Hospital, NHO Tokyo Medical Center, and Nagasaki University Hospital. Patient recruitment and follow-up were conducted between April 2020 and March 2025, from the first patient's first visit (September 11, 2020) to the last patient's last visit (September 18, 2024).

The study was conducted in accordance with the Declaration of Helsinki and was approved by the institutional review boards of all participating centers. Written informed consent was obtained from all participants prior to enrollment.

### Participants

Patients were enrolled from 6 centers according to predefined inclusion/exclusion criteria. Inclusion criteria were as follows: (1) presence of biallelic pathogenic or likely pathogenic variants in the *CYP4V2* gene; (2) age ≥20 years at the time of enrollment; and (3) BCVA of 1.0 logMAR or better in ≥1 eye at baseline.

Exclusion criteria included the presence of other retinal diseases, significant media opacity (e.g., dense cataract or vitreous hemorrhage) that interfered with image acquisition, poor fixation, or history of ocular surgery within 6 months prior to enrollment.

A total of 13 patients (26 eyes) were enrolled. Follow-up assessments were conducted at 6-month intervals, consistent with the standard practice for patients with inherited retinal degeneration, to enable reliable detection of disease progression while ensuring feasibility for long-term follow-up. No patient was lost to follow-up during the study period.

### Endpoints

The primary endpoint of this study was the change in BCVA over the follow-up period. Secondary endpoints included changes in MD on Humphrey 10-2 visual field testing, central subfield thickness (CST) on OCT, and the area of RPE atrophy on fundus autofluorescence (FAF) images.

### Clinical Examination

Comprehensive ophthalmic examinations, including BCVA, static perimetry, OCT, and detailed funduscopic examination, were conducted according to the procedures described here.

Best-corrected visual acuity was initially measured on the decimal scale using Landolt C charts (CV-6000 or AVC-36) at a 5-m distance. Both devices used the same series of Landolt C optotypes and identical decimal acuity steps; therefore, the BCVA measurements were directly comparable across centers. All visual acuity examinations were performed by well-trained certified orthoptists. The decimal values were converted into logMAR units for statistical analyses. The logMAR values for counting fingers, hand movements, light perception, and no light perceptions were defined as 2.3 logMAR, 2.6 logMAR, 2.9 logMAR, and 3.2 logMAR, respectively.^23^

Refractive error was measured using a subjective refraction test at baseline. The spherical equivalent was calculated as the spherical power plus half of the cylindrical power (Table S1, available at www.ophthalmologyscience.org).

Visual field testing was performed using automated static perimetry with the Humphrey Field Analyzer (Humphrey Instruments), employing the 10-2 Swedish Interactive Thresholding Algorithm Standard protocol. Only tests meeting reliability criteria (fixation loss ≤20%, false positives ≤15%, and false negatives ≤33%) were included. Three visual field parameters were extracted for analysis: MD, absolute scotoma point (ASP), and transitional zone retinal sensitivity (TZRS). Mean deviation is a global index that represents the difference between a patient's visual field sensitivity and that of age-matched controls and is automatically calculated by the Humphrey Field Analyzer software. Absolute scotoma point refers to the number of test points among the 68 examined locations that show a retinal sensitivity of 0 dB. For TZRS analysis, transitional zone was defined as the loci surrounding a location with 0 dB sensitivity on baseline perimetry (Fig. S1, available at www.ophthalmologyscience.org), and the averaged retinal sensitivity within the TZ on baseline was defined as TZRS. The same region was tracked across follow-up visits to calculate TZRS at each visit (Fig. S1).

Color fundus photography was obtained using a fundus camera (Topcon) or the Optos ultra-widefield imaging device (Nikon). Near-infrared reflectance imaging was performed with the infrared reflectance mode of the Spectralis HRA+OCT system (Heidelberg Engineering). Images were acquired using an 820 nm infrared laser and a 55° field of view. OCT of the macula was obtained using the same Spectralis HRA+OCT platform (Heidelberg Engineering). All OCT B-scans were acquired in high-speed mode. Horizontal and vertical line scans (9 mm in length) centered on the fovea were acquired with automatic real-time averaging of 40 to 60 frames. Central subfield thickness was manually measured on the foveal B-scan. A vertical line perpendicular to the RPE was drawn at the center of the fovea, and the distance between the inner limiting membrane and the outer border of the RPE was recorded as the CST. When an epiretinal membrane was present, the measurement included the epiretinal membrane thickness. For eyes imaged with macular analysis mode, the central foveal slice was used; if only horizontal and vertical scans were available, the horizontal foveal scan was selected.

Fundus autofluorescence images were acquired using the Spectralis HRA+OCT system (Heidelberg Engineering) with the Heidelberg Eye Explorer software (version 1.10.2.0). Images were obtained using the BluePeak autofluorescence mode with a 55° field of view, employing a blue excitation light of 486–488 nm and detecting emitted light through a barrier filter transmitting wavelengths >500 nm. The images were captured at a resolution of 768 × 768 pixels, with an automatic real-time averaging of 20–30 frames. The hypo-autofluorescent (hypo-AF) areas were defined as regions with definitely decreased autofluorescence intensity comparable to that of the optic nerve head and retinal blood vessels, as described in the ProgStar study evaluating the progression of Stargardt disease.^24^ These areas were measured manually using ImageJ, and pixel areas were converted to mm^2^ based on the embedded scale bar. The hypo-AF area was quantified within the central 55° FAF field. Eyes with baseline hypo-AF areas occupying >55% of the 55° field were excluded because further enlargement could not be reliably assessed within the measurable field. Eyes without a detectable hypo-AF area at baseline were also excluded. When the hypo-AF area extended beyond the 55° field, the outer boundary of the image was defined as the border of the hypo-AF area. If the hypo-AF region encroached with areas of peripapillary atrophy, the peripapillary atrophy area was included in the measurement. Baseline and last-visit measurements of hypo-AF areas in all included eyes are shown in Figure S4 (available at www.ophthalmologyscience.org). Measurements of hypo-AF areas were performed by 2 trained graders (H.Z. and S.S.) in accordance with a measurement protocol supervised by the principal investigator (Y.M.).

### Statistical Analyses

Linear mixed-effects models were employed to assess longitudinal structural and functional changes, as well as their interrelationships, while accounting for repeated measures within subjects and intereye correlation. First, to quantify disease progression, separate linear mixed-effects models were constructed for each outcome variable, such as hypo-AF area, BCVA, MD, ASP, TZRS, and CST, with visit time as a fixed effect. The estimated coefficients represent the annual rate of change for each parameter. For each parameter, we calculated the annual change (slope; units/year), annual percentage change (%/year), residual standard deviation (SD), minimal detectable change at 95% confidence, and signal-to-noise ratio (SNR = |slope|/residual SD). Residual SD represents the variability of data points around the fitted slope; a smaller residual SD indicates better model fit and less measurement noise. Minimal detectable change at 95% confidence is the smallest change that can be interpreted as a real change with 95% confidence; smaller minimal detectable change at 95% confidence values indicate higher sensitivity for detecting progression. Signal-to-noise ratio reflects the magnitude of the observed change relative to variability; higher SNR values indicate a clearer signal of change over time. Second, to evaluate the association between structural and functional progression, additional linear mixed-effects models were constructed using the slope of each functional parameter (BCVA, MD, ASP, TZRS, and CST) as the dependent variable and the slope of hypo-AF area after applying square root transformation prior to slope estimation as the fixed-effect predictor. To assess the repeatability/reproducibility of hypo-AF area measurements, the intraclass correlation coefficient was calculated using a 2-way random-effects model. In all models, random interceptions were specified for eyes nested within patients to adjust for intrasubject and intereye variability. Statistical analyses were conducted in R (version 2024.12.1+563, R Foundation for Statistical Computing) using the lme4, lmerTest, emmeans, and tidyverse packages. A 2-sided *P* value <0.05 was considered statistically significant.

## Results

### Patient Characteristics

A total of 13 patients (4 males and 9 females) with BCD were enrolled. Table 1 summarizes the patient characteristics. The median age at first visit was 66 years (range: 43–75 years), and the median follow-up was 2.5 years (range: 2.5–3 years). All patients were confirmed to carry biallelic pathogenic variants in the *CYP4V2* gene, with recurrent variants including c.802-8_810delinsGC, c.1020G>A (p.Trp340Ter), and c.518T>G (p.Leu173Trp).

**Table 1:** Genetic and Clinical Features of 13 Patients with Bietti Crystalline Dystrophy

Patient Number	Sex	Nucleotide Change	Protein Change	Zygosity	Follow-up (Yrs)	Age at First Visit (Yrs)
OPH-728	F	c.518T>G	p.Leu173Trp	Homozygous	3	77
OPH-207	F	c.810delT	p.Glu271AsnfsTer6	Homozygous	2.5	65
OPH-437	M	c.518T>G	p.Leu173Trp	Homozygous	2.5	56
OPH-330	M	c.1199G>A	p.Arg400His	Homozygous	2.5	56
OPH-990	F	c.802-8_810delinsGC	p.V268_E329del	Homozygous	2.5	55
OPH-219	F	c.802-8_810delinsGC	p.V268_E329del	Homozygous	2.5	66
OPH-227	F	c.1020G>A	p.Trp340Ter	Homozygous	3	74
OPH-325	F	c.1020G>A	p.Trp340Ter	Homozygous	2.5	72
BT-001	M	c.802-8_810delinsGC	p.V268_E329del	Homozygous	3	47
BT-002	F	c.802-8_810delinsGC	p.V268_E329del	Homozygous	2.5	73
K1	F	c.802-8_810delinsGC	p.V268_E329del	Homozygous	2.5	43
K2	M	c.327+1G>A/c.802-8_810del17insGC	Splice site alteration/p.V268_E329del	Compound heterozygous	3	69
K3	F	c.1199G>A/c.802-810del17insGC	p.Arg400His/p.V268_E329del	Compound heterozygous	3	69

F = female; M = male.

Transcript ID: NM_207352.4.

Refractive error data were available for 26 eyes from 13 patients, with a mean ± SD spherical equivalent of –1.80 ± 1.79 D (Table S1).

### The Clinical Features and Progression of BCD

The characteristic pathological features of BCD were clearly demonstrated across multimodal imaging modalities (Fig. S2, available at www.ophthalmologyscience.org). Color fundus photography revealed numerous glistening yellow–white crystalline deposits in the posterior pole and mid-periphery. These corresponded to hyperreflective puncta on near-infrared reflectance–FAF, indicating their spatial distribution. Short-wavelength FAF showed a sharply demarcated central hypo-AF area, consistent with RPE atrophy. The intraclass correlation coefficient for hypo-AF area measurements between 2 graders (H.Z. and S.S.) was 0.999, indicating very high reproducibility; therefore, H.Z.'s measurements were used for the analyses. Spectral-domain OCT demonstrated hyperreflective crystalline material in the RPE, associated with retinal thinning and ellipsoid zone disruption.

**Figure 1:**
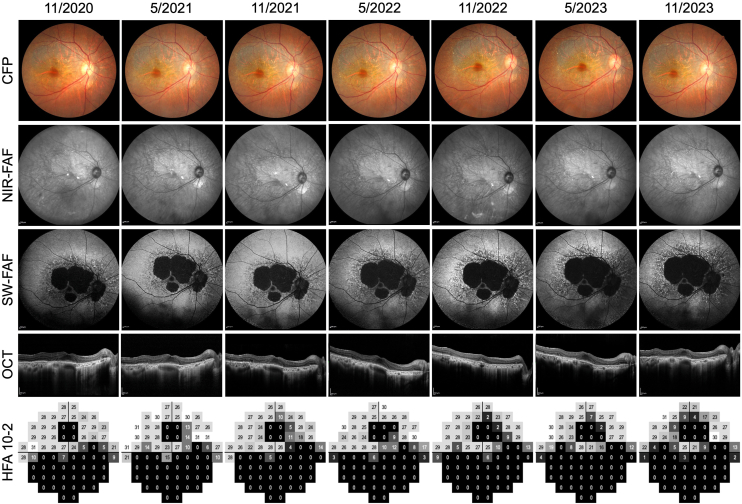
Longitudinal structural and functional changes in a representative BCD case (case 1). From November 2020 to November 2023, CFP shows crystalline deposits and RPE atrophy; NIR-FAF highlights crystals; SW-FAF shows progressive hypo-AF enlargement; OCT reveals outer retinal thinning and EZ loss; and HFA 10-2 indicates parafoveal sensitivity loss. BCD = Bietti crystalline dystrophy; CFP = color fundus photography; EZ = ellipsoid zone; FAF = fundus autofluorescence; HFA = Humphrey Field Analyzer; hypo-AF = hypo-autofluorescent; NIR = near-infrared reflectance; RPE = retinal pigment epithelium; SW-FAF = short-wavelength fundus autofluorescence.

Figure 1 presents the longitudinal clinical course of case 1 with >6 visits. Progressive enlargement of the hypo-AF area was observed on short-wavelength FAF, along with gradual outer retinal atrophy on OCT. Correspondingly, retinal sensitivity in Humphrey Field Analyzer 10-2 tests gradually declined, accompanied by scotoma expansion.

**Figure 2:**
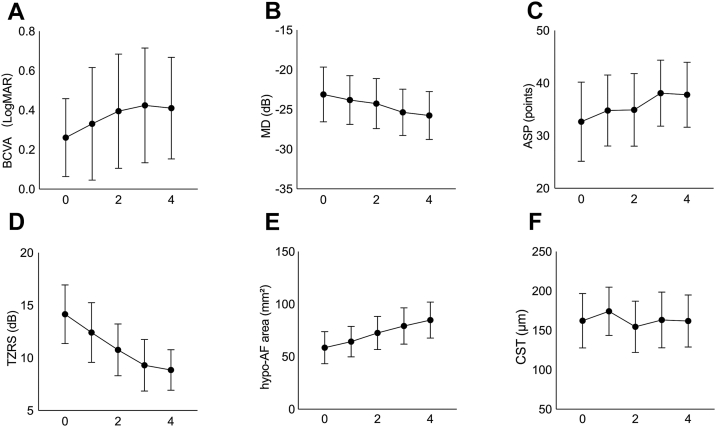
Longitudinal changes in visual function and structural parameters. Mean ± 95% CI values at each visit are shown for: **(A)** BCVA (logMAR), **(B)** MD (dB), **(C)** ASP (points), **(D)** TZRS (dB), **(E)** hypo-AF area (mm^2^), and **(F)** CST (μm). ASP = absolute scotoma point; BCVA = best-corrected visual acuity; CI = confidence interval; CST = central subfield thickness; hypo-AF = hypo-autofluorescent; logMAR = logarithm of the minimum angle of resolution; MD = mean deviation; TZRS = transitional zone retinal sensitivity.

Baseline and last follow-up values for all functional and structural variables are summarized in Table 2 as median with interquartile ranges. Figure 2 and Figure S3 (available at www.ophthalmologyscience.org) demonstrate the longitudinal changes of BCVA, MD, ASP, TZRS, CST, and hypo-AF area, respectively, in patients with BCD. Linear mixed-effects models revealed a significant annual increase in the hypo-AF area (12.0 mm^2^/year, 95% confidence interval [CI]: 7.1–16.9, *P* = 0.003) and ASP (2.4 points/year, 95% CI: 1.8–3.0, *P* < 0.001), a significant decline in MD (–1.0 dB/year, 95% CI: –1.7 to –0.3, *P* = 0.02), TZRS (–2.1 dB/year, 95% CI: –2.8 to –1.4, *P* < 0.001), CST (–4.3 μm/year, 95% CI: –6.6 to –2.1, *P* < 0.001), and BCVA (0.04 logMAR/year, 95% CI: 0.01–0.07, *P* = 0.01; Table 3). Linear mixed-effects modeling of √hypo-AF area also showed a significant annual increase (0.7 √mm²/year, 95% CI: 0.6–0.9, *P* < 0.001; Table S2, available at www.ophthalmologyscience.org). One patient (K2) was excluded from the static perimetry analyses because test intervals exceeded 6 months. In addition, 1 eye (right eye, K3) was excluded from ASP and TZRS because no zero sensitivity points were present at baseline. Consequently, 24 eyes were included in the MD analysis and 23 eyes in the ASP and TZRS analyses. For hypo-AF area analysis, 12 eyes of 6 patients were excluded, leaving 14 eyes for analysis.

**Table 2:** Baseline and Last Follow-up Measurements of Functional and Structural Parameters

Variable∗	Baseline (Median [IQR])	Last Follow-up (Median [IQR])
BCVA (logMAR)	0.03 (–0.08 to 0.30)	0.19 (0.05 to 0.82)
MD (dB)	–25.1 (–29.5 to –18.5)	–27.2 (–31.2 to –23.1)
ASP (points)	33.0 (20.0 to 46.5)	36.0 (29.0 to 48.0)
TZRS (dB)	13.6 (9.0 to 19.0)	9.3 (5.4 to 11.3)
CST (μm)	168.0 (94.8 to 237.5)	142.5 (87.3 to 231.3)
Hypo-AF area (mm^2^)	64.0 (33.0 to 79.5)	88.6 (61.7 to 110.8)

ASP = absolute scotoma point; BCVA = best-corrected visual acuity; CST = central subfield thickness; hypo-AF area = hypo-autofluorescent area; IQR = interquartile range; logMAR = logarithm of the minimum angle of resolution; MD = mean deviation; TZRS = transitional zone retinal sensitivity.

∗ The number of eyes included in the analyses are as follows: n = 26 eyes for BCVA and CST; n = 24 for MD; n = 23 for ASP and TZRS; n = 14 for hypo-AF area.

**Table 3:** Progression Rates of Retinal Parameters, Variability, and Ability to Detect Progression Using Univariate LMMs

Variable∗	Coefficient	95% CI	*P* Value	Residual SD	Annual Change (%)	SNR	MDC95
BCVA (logMAR/yr)	0.04	0.01 to 0.07	**0.01**	0.25	7.6	0.14	0.7
MD (dB/yr)	–1.0	–1.7 to –0.3	**0.02**	1.9	–4.1	0.53	5.4
ASP (points/yr)	2.4	1.8 to 3.0	**<0.001**	5.4	7.2	0.44	15.0
TZRS (dB/yr)	–2.1	–2.8 to –1.4	**<0.001**	2.7	–15.7	0.78	7.6
CST (μm/yr)	–4.3	–6.6 to –2.1	**<0.001**	12.6	–2.7	0.33	34.9
Hypo-AF area (mm^2^/yr)	12.0	7.1 to 16.9	**0.003**	11.1	21.6	1.10	30.9

ASP = absolute scotoma point; BCVA = best-corrected visual acuity; CI = confidence interval; CST = central subfield thickness; hypo-AF area = hypo-autofluorescent area; LMMs = linear mixed models; logMAR = logarithm of the minimum angle of resolution; MD = mean deviation; MDC95 = minimal detectable change at 95% confidence; SD = Stargardt disease; SNR = signal-to-noise ratio; TZRS = transitional zone retinal sensitivity.

*P* < 0.05 was considered statistically significant.

Bold values indicate statistical significance.

∗ The number of eyes included in the analyses are as follows: n = 26 eyes for BCVA and CST; n = 24 for MD; n = 23 for ASP and TZRS; n = 14 for hypo-AF area.

Table 3 summarizes the progression rate, variability, and ability of the 5 retinal parameters to detect progression. Among the 14 eyes analyzed, the hypo-AF area showed the largest annual change rate (21.6%) and the highest SNR (1.10). Transitional zone retinal sensitivity exhibited the second highest SNR (0.78) despite a smaller slope, while BCVA showed minimal progression with the lowest SNR (0.14).

### Relationship between Structural and Functional Disease Progression

**Figure 3:**
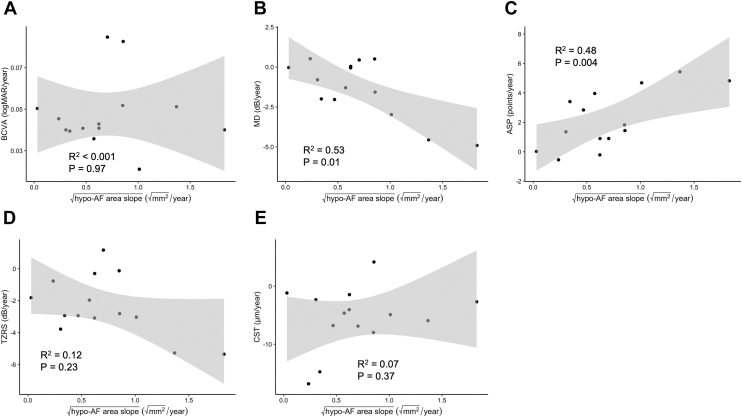
Associations between the yearly slope of √hypo-AF area and functional or structural parameters. Scatterplots show the relationships between √hypo-AF area progression and annual changes in **(A)** BCVA, **(B)** MD, **(C)** ASP, **(D)** TZRS, and **(E)** CST. Ninety-five percent confidence shading are shown; *R*^*2*^ and *P* values are from simple linear regression. ASP = absolute scotoma point; BCVA = best-corrected visual acuity; CST = central subfield thickness; hypo-AF = hypo-autofluorescent; logMAR = logarithm of the minimum angle of resolution; MD = mean deviation; TZRS = transitional zone retinal sensitivity.

Next, the association between structural and functional progression in BCD was assessed. Linear mixed-effects modeling revealed significant associations between the progression of square root hypo-AF area and changes in MD (estimate: –2.6 dB/mm; 95% CI: –4.1 to –1.2; *P* = 0.01) and ASP (estimate: 2.9 points/mm; 95% CI: 1.2–4.5; *P* = 0.004). In contrast, the associations with changes in BCVA (estimate: 0.003 logMAR/mm; 95% CI: –0.02 to 0.02; *P* = 0.97), TZRS (estimate: –1.3 dB/mm; 95% CI: –3.3 to 0.7; *P* = 0.23), and CST (estimate: 2.7 μm/mm; 95% CI: –2.9 to 8.3; *P* = 0.37) did not reach statistical significance (Table 4, Fig. 3).

**Table 4:** Association between Progression in Square Root-Transformed Hypo-AF Area and Visual Functional Parameters Analyzed by LMM

Variable∗	Estimate†	Std. Error	95% CI	*P* Value
BCVA (logMAR/yr)	0.003	0.01	–0.02 to 0.02	0.97
MD (dB/yr)	–2.6	0.8	–4.1 to –1.2	**0.01**
ASP (points/yr)	2.9	0.8	1.2 to 4.5	**0.004**
TZRS (dB/yr)	–1.3	1.0	–3.3 to 0.7	0.23
CST (μm/yr)	2.7	2.9	–2.9 to 8.3	0.37

ASP = absolute scotoma point; BCVA = best-corrected visual acuity; CI = confidence interval; CST = central subfield thickness; hypo-AF area = hypo-autofluorescent area; LMM = linear mixed model; logMAR = logarithm of the minimum angle of resolution; MD = mean deviation; TZRS = transitional zone retinal sensitivity.

*P* < 0.05 was considered statistically significant.

Bold values indicate statistical significance.

∗ All variables were analyzed using the same 14 eyes.

† The unit of the estimate was defined as the unit of the slope for each variable divided by (√mm^2^/yr). For example, the unit of the estimate for BCVA was (logMAR/yr)/(√mm^2^/yr).

## Discussion

This prospective multicenter study investigated the natural history of BCD. There were 2 main findings. (1) Over the follow-up period (median 2.5 years), significant progression was observed in the BCVA, MD, ASP, TZRS, CST, and hypo-AF area. (2) The hypo-AF area progression was significantly associated with the loss of visual function, supporting its use as a useful biomarker of BCD progression.

*CYP4V2* is highly expressed in the RPE, and dysfunction and degeneration of the RPE are considered the primary pathological mechanisms of BCD.^4^^,^^8^ Accordingly, FAF imaging, which enables noninvasive assessment of RPE integrity, plays a central role in both the diagnosis and monitoring of BCD.^25^, ^26^, ^27^, ^28^, ^29^ Previous studies have reported that FAF abnormalities, particularly hypo-AF areas, reflect underlying RPE degeneration that may precede photoreceptor loss and visual field defects in BCD patients.^21^^,^^30^^,^^31^ In this prospective study, we demonstrated that among all parameters evaluated, the hypo-AF area exhibited the most statistically significant longitudinal change, with a mean annual expansion of approximately 12.0 mm^2^. This increase was consistently observed even in patients whose BCVA remained relatively stable, underscoring its utility as a sensitive biomarker for monitoring disease progression in BCD.

In dry age-related macular degeneration (AMD), a condition also characterized by primary RPE degeneration, FAF-based endpoints such as geographic atrophy enlargement have been adopted as a primary outcome measure in clinical trials.^32^, ^33^, ^34^ Dry AMD and BCD share several pathological features, including progressive RPE atrophy, outer retinal tubulation, and secondary photoreceptor degeneration.^35^, ^36^, ^37^ Fundus autofluorescence-based endpoints have also been applied in Stargardt disease, with progression of definitely decreased autofluorescence on FAF being reported as 0.74 mm^2^/year (95% CI: 0.64–0.85; *P* < 0.0001). Fundus autofluorescence progression has been used as a primary outcome measure in Stargardt disease clinical trials and may help track disease progression.^24^^,^^38^ Given the overlap in pathology and its correlation with visual function, the hypo-AF area may represent a potential outcome measure in future clinical trials for BCD.

Retinal sensitivity changes in the transitional zone between preserved and atrophic retinal areas (termed the TZRS in this study) have attracted attention as a sensitive measure for detecting disease progression.^39^ For instance, mesopic TZRS measurements in the junctional zones of geographic atrophy decrease sharply in AMD patients prior to functional loss.^39^ In retinitis pigmentosa, the PREP-1 study showed that regional TZRS declines significantly, even when visual acuity and fixation remain stable, highlighting its utility for detecting early functional change.^40^ In our BCD cohort, progression rates for the TZRS area exceeded those for BCVA, MD, CST, and ASP, underscoring the ability of TZRS to sensitively capture early functional decline. Transitional zone retinal sensitivity, however, also exhibited considerable within-patient variability across visits. The hypo-AF area showed relatively consistent progression across patients (Fig. S3). This aligns with the retinitis pigmentosa and AMD literature: although TZRS-based metrics are sensitive to early dysfunction, they are susceptible to test–retest variability, measurement noise, and subject factors. Refractive error may influence retinal area measurements through magnification effects,^41^ but this impact is considered negligible in our within-eye longitudinal analyses.

A positive coefficient between hypo-AF area enlargement and increasing CST (2.7 μm/year; Table 4) was an unexpected finding, although this association did not reach statistical significance (*P* = 0.37). One possible explanation is the spatial discrepancy between these parameters: CST reflects retinal thickness within the central 1-mm macular area, whereas the hypo-AF area represents atrophy across a broader 55° field. In BCD, atrophy often progresses in the mid-periphery, while the fovea remains relatively preserved (“foveal sparing”) until the later stages. In such cases, rapid expansion of the hypo-AF area may occur, while CST remains stable, potentially skewing the coefficient toward a positive direction. Second, CST measurements may show some variability due to slight differences in OCT scan location between visits. Third, the development of epiretinal membrane during follow-up may also influence CST measurements, as tractional effects can lead to central retinal thickening.

There were several limitations in this study. First, the small sample size limited the statistical power and sensitivity to detect subtle changes in visual parameters such as BCVA, as well as to analyze the genotype–phenotype relationships. Second, the cohort had a relatively high mean age, and most patients were at advanced stages. Although age may influence the progression rate of BCD, this factor could not be evaluated due to the limited sample size. Third, the ethnic homogeneity of the cohort, which consisted exclusively of Japanese patients, may reduce the generalizability of our findings to diverse BCD populations. Fourth, although previous studies have demonstrated the utility of quantifying crystalline deposits,^42^ outer retina tubulation,^43^ and ellipsoid zone length on OCT,^36^ these parameters were not evaluated in this study due to limitations in quantification reliability and variability in image quality. Fifth, although eyes with extensive involvement of the 55° field by the hypo-AF area were excluded; in some cases, a portion of the hypo-AF area extended beyond the imaging field. A sufficient area of relatively preserved retina remained to permit assessment of progression in these eyes; however, the progression rate may have been underestimated. These points should be addressed in future research.

In conclusion, hypo-AF area is a sensitive measure of BCD progression, showing a strong correlation with visual decline.
