# Supplementary material for: Fundus Autofluorescence as a Sensitive Biomarker of Disease Progression in Bietti Crystalline Dystrophy
Source: Ophthalmol Sci. 2026 Mar 19;6(5):101166. doi: 10.1016/j.xops.2026.101166 (PMC13096951; doi:10.1016/j.xops.2026.101166)
Supplement: Figure S1 [file mmc1.pdf]

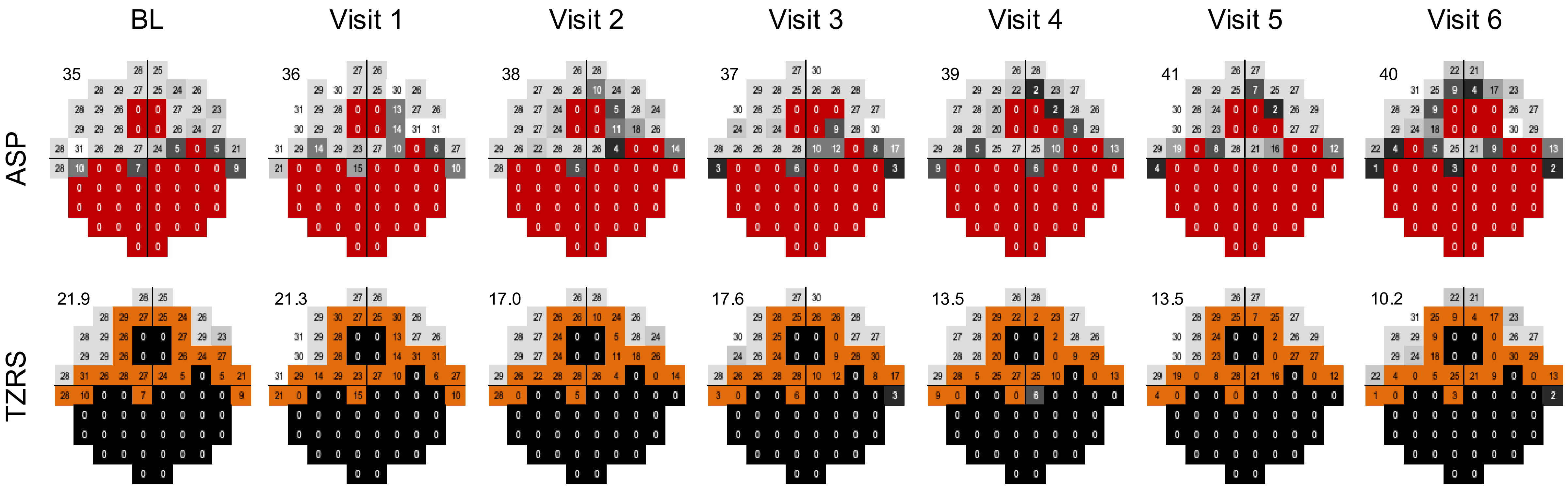

Figure S1. Definition of ASP and TZRS on HFA 10-2 visual field testing across seven visits (OPH-728).

ASP (red) includes all test points with 0 dB sensitivity. TZRS includes all surrounding points with non-zero sensitivity around the ASP. Upper panels show ASP; lower panels show corresponding TZRS at each time point.
