# Supplementary material for: Fundus Autofluorescence as a Sensitive Biomarker of Disease Progression in Bietti Crystalline Dystrophy
Source: Ophthalmol Sci. 2026 Mar 19;6(5):101166. doi: 10.1016/j.xops.2026.101166 (PMC13096951; doi:10.1016/j.xops.2026.101166)
Supplement: Figure S2 [file mmc2.pdf]

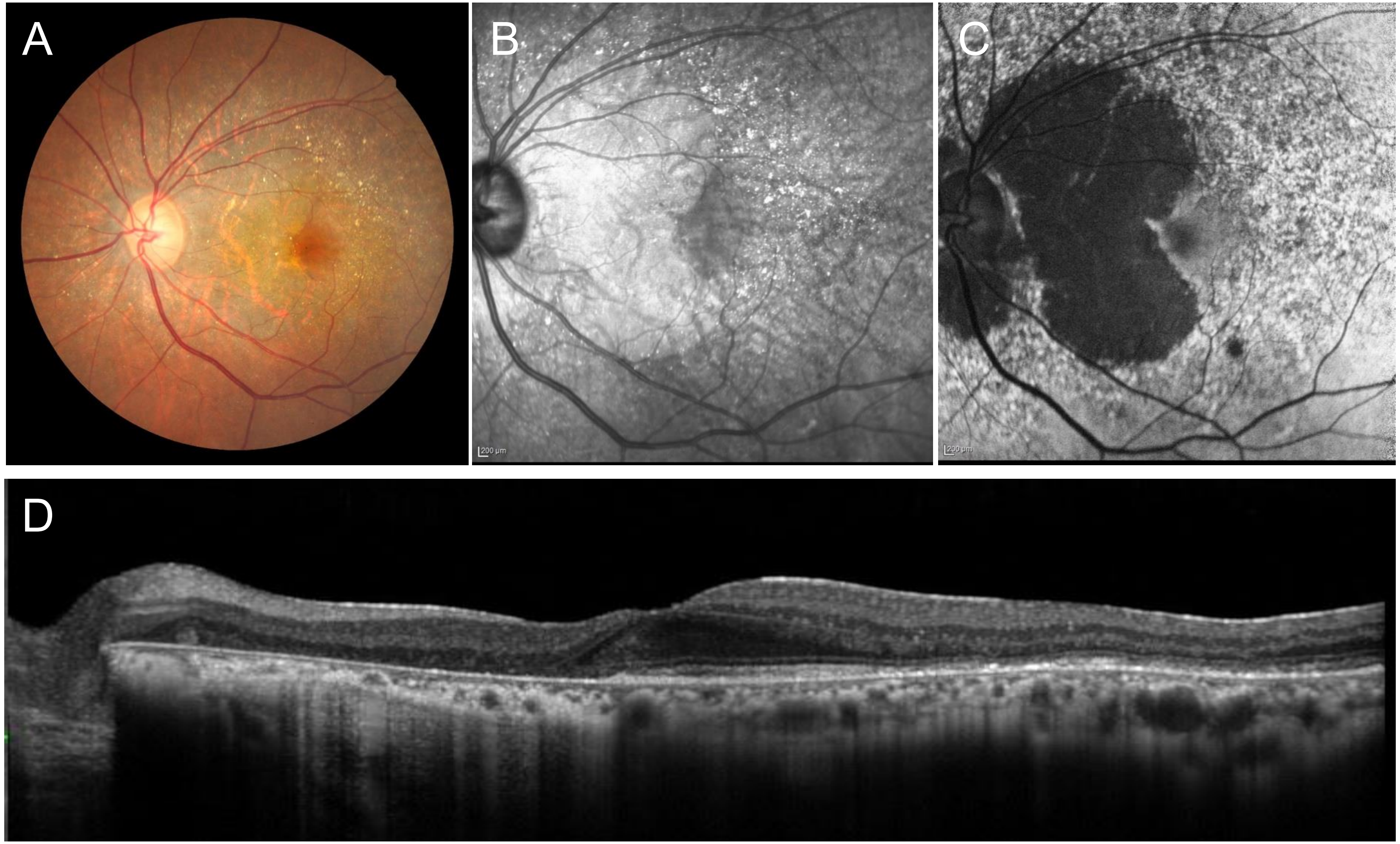

Figure S2. Representative multimodal imaging features of BCD.

(A) CFP showing numerous yellow-white crystalline deposits in the posterior pole and mid-periphery. (B) NIR-FAF highlighting hyperreflective crystalline deposits and hypo-AF areas corresponding to RPE atrophy. (C) SW-FAF revealing extensive hypo-AF regions indicative of RPE degeneration. (D) OCT demonstrating outer retinal disruption and hyperreflective crystalline material at or above the RPE.
