# Supplementary material for: Fundus Autofluorescence as a Sensitive Biomarker of Disease Progression in Bietti Crystalline Dystrophy
Source: Ophthalmol Sci. 2026 Mar 19;6(5):101166. doi: 10.1016/j.xops.2026.101166 (PMC13096951; doi:10.1016/j.xops.2026.101166)
Supplement: Figure S3 [file mmc3.pdf]

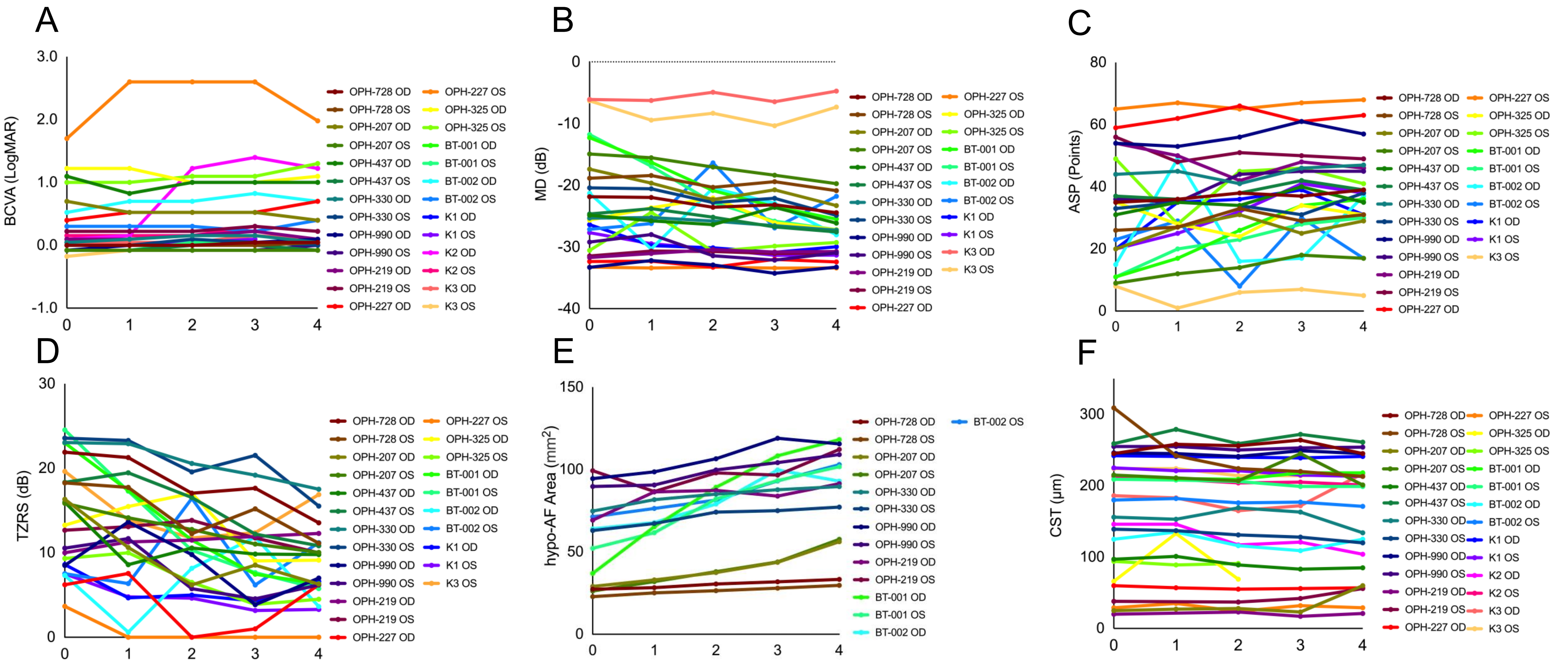

Figure S3. Individual trajectories and estimated annual changes in visual and structural parameters.

(A–E) Longitudinal changes of individual eyes for BCVA (A), MD (B), ASP (C), TZRS (D), hypo-AF area (E) and CST (F). Each colored line represents one eye.
