# Supplementary material for: Fundus Autofluorescence as a Sensitive Biomarker of Disease Progression in Bietti Crystalline Dystrophy
Source: Ophthalmol Sci. 2026 Mar 19;6(5):101166. doi: 10.1016/j.xops.2026.101166 (PMC13096951; doi:10.1016/j.xops.2026.101166)
Supplement: Figure S4 [file mmc4.pdf]

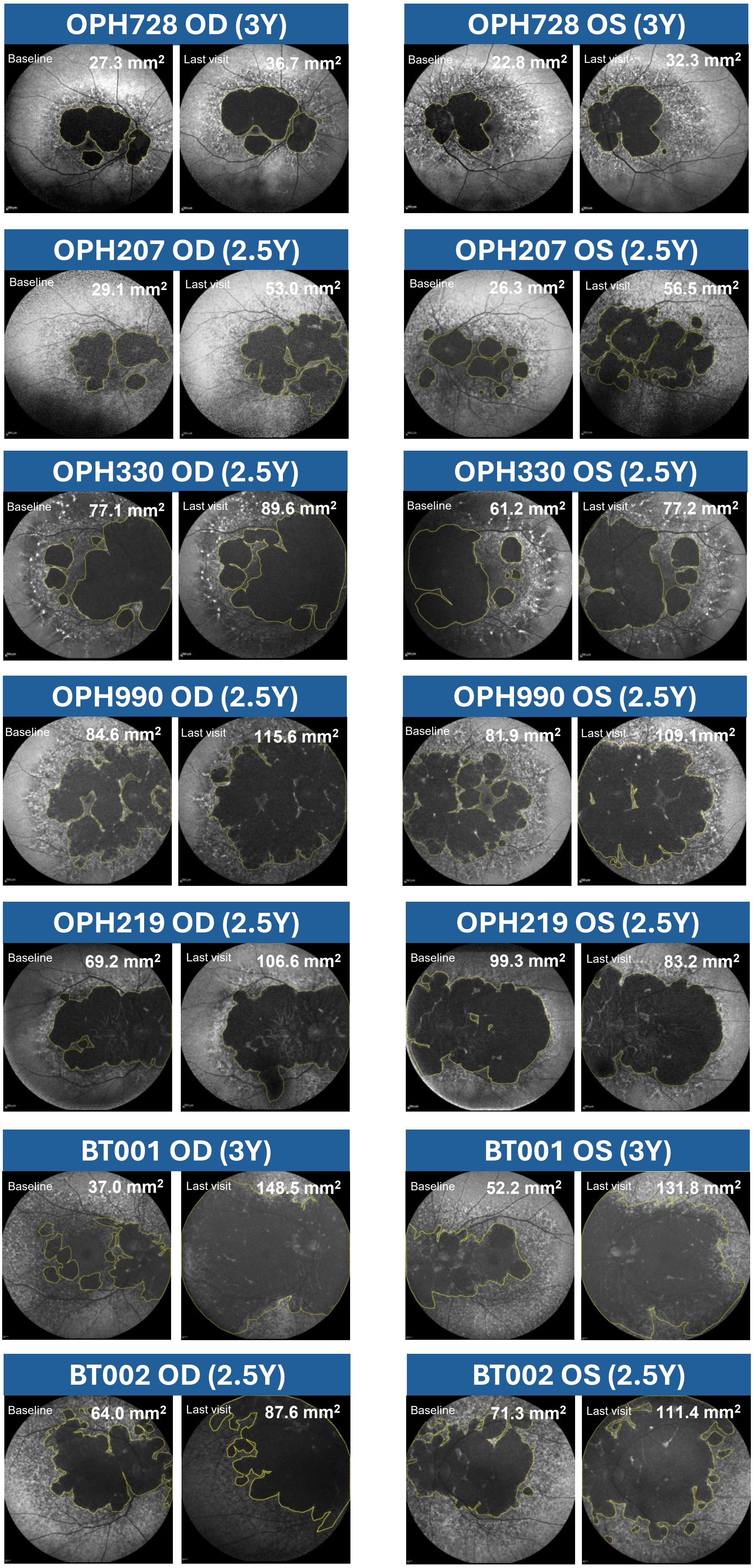

Figure S4. Longitudinal measurement of hypo-AF area in all included eyes. Baseline and last-visit central 55° FAF images of all 14 eyes (7 patients) included in the hypo-AF area analysis. The manually delineated hypo-AF areas and corresponding measured values (mm^2) are shown for each eye.
