# Supplementary material for: Fundus Autofluorescence as a Sensitive Biomarker of Disease Progression in Bietti Crystalline Dystrophy
Source: Ophthalmol Sci. 2026 Mar 19;6(5):101166. doi: 10.1016/j.xops.2026.101166 (PMC13096951; doi:10.1016/j.xops.2026.101166)
Supplement: Table S1 [file mmc5.pdf]

Table S1. Refractive status of the patients

| Patient ID | SE |
|---|---|
| OPH-728 OD | −0.75 |
| OPH-728 OS | −0.75 |
| OPH-207 OD | −5.63 |
| OPH-207 OS | −3.00 |
| OPH-437 OD | −4.50 |
| OPH-437 OS | −3.25 |
| OPH-330 OD | 0.00 |
| OPH-330 OS | 0.00 |
| OPH-990 OD | −1.50 |
| OPH-990 OS | −1.50 |
| OPH-219 OD | 0.00 |
| OPH-219 OS | −0.50 |
| OPH-227 OD | −3.00 |
| OPH-227 OS | −3.75 |
| OPH-325 OD | 0.75 |
| OPH-325 OS | 2.00 |
| K1 OD | −3.25 |
| K1 OS | −2.63 |
| K2 OD | −3.88 |
| K2 OS | −3.63 |
| K3 OD | −2.38 |
| K3 OS | −2.13 |
| BT-001 OD | −1.00 |
| BT-001 OS | −1.00 |
| BT-002 OD | −0.75 |
| BT-002 OS | −0.75 |

SE= Spherical Equivalent
