# Supplementary material for: Fundus Autofluorescence as a Sensitive Biomarker of Disease Progression in Bietti Crystalline Dystrophy
Source: Ophthalmol Sci. 2026 Mar 19;6(5):101166. doi: 10.1016/j.xops.2026.101166 (PMC13096951; doi:10.1016/j.xops.2026.101166)
Supplement: Table S2 [file mmc6.pdf]

Table S2. Comparison of progression for hypo-AF area and √hypo-AF area based on LMMs

Model*	Coefficient	95% CI	P value
hypo-AF area (mm^2/year)	12.0	7.1 to 16.9	**0.003**
√hypo-AF area (√mm^2/year)	0.7	0.6 to 0.9	**< 0.001**

LMMs = Linear mixed-effects models; BCVA = best-corrected visual acuity; $P < 0.05$ was considered statistically significant. *All analyses were performed using 14 eyes.
